# Cleansing the Air

**Published:** 1920-03-27

**Authors:** 


### THE PUBLIC WELL-BEING.

### CLEANSING THE AIR.

*[The Minister of Health has appointed a Committee to consider the present state of the law with regard to the pollution of the air by smoke and other noxious vapours, and to advise what steps are desirable and practicable to diminish the evils still arising from such pollution.]*

Anyone living in an industrial town who has walked through its streets just after dawn on a fine day must have felt the subtle difference in the air from the conditions of the later part of the day. But, since self-analysis puts a brake on the enjoyment of such moments, it is very possible one has not paused to ask what causes and constitutes this difference. It has sufficed that there has been a newness about old and familiar things; the eye has travelled more easily and with greater pleasure along the vista of shops and houses; the air has braced the lungs; and the sky has been brighter and clearer, seen without its working-day veil. Perhaps when in the same streets at noon on a fine Sunday, the wheels of industry being stilled, or in some non-industrial continental or home town, we have found much the same conditions reproduced, except for the thrill of dawn. We have had some faint glimmering of the harm to the senses and to the health that air pollution by smoke involves.

At this moment it is impossible to calculate whether the war has advanced or retarded the movement for smoke abatement. One hears little at present which seems so hopeful as were the investigations which were started in sixteen large cities a little before the outbreak of war to determine scientifically the nature and amount of impurities in the air; but absence of news may be due to the fact of the diversion of energy from the work of compiling reports and not from less energetic inquiry. Much of the impulse in those days came from local enthusiasts whose attention in the war had to be given to more pressing matters, but the Meteorological Office, which continued the investigations, and the Departmental Committee appointed after the introduction of Lord Newton's Bill on smoke abatement early in 1914, may yet show that time apparently lost has been used with some benefit. If, however, we concede that the war reduced both the interest and effort devoted to air sanitation, we have on the credit side the great schemes for electrical power production, which will when realised very largely reduce industrial smoke pollution, and the impulse to communal effort which is helping forward schemes for central hot-water systems for working-class houses, one object of which is to reduce domestic smoke.

The illustration at the beginning of this article was given advisedly. For it is an example of seeing the smoke evil by direct observation. Examples of vicarious observation, more exact but less impressive, could be multiplied to show how exceedingly serious the amount of air pollution in industrial areas is: for instance, in Manchester it was calculated that in July 1914—results vary according to the month—a circle of a mile radius with its centre near the centre of the city would have yielded 195 tons of air impurities, 100 tons of which would have consisted of soot or other insoluble matter. So it is found that rain water collected in an industrial section of that circle is thick and dark with impurities, while rain water from a Cheshire suburb of the city is almost of natural colour and consistency. And one might add many other striking results of experiment if there were space; but one would rather say, let the reader try for himself the single experiment of seeing his own town when the production of smoke is at a minimum and contrasting the conditions of the working day.

Now, what are the effects of smoke pollution? We will not stress the remarkable fact that in the report recently issued of the medical surveys made during the war the worst physique was found precisely in the areas where most air pollution is found. Can there be any question, however, that there must be some connection between the two, especially in view of the frequency of lung trouble? But here, again, one would call in the aid of direct observation. Any dweller in a smoky town knows the difference a spell in clean air—a game of golf, a walk over moors, or a turn at the seaside—makes to his spirits and health. Discounting all other factors—the relief from mental strain, the tonic of sea and moor—is there not a lot left that is simply and solely due to the fact that one has breathed air which carries no burden of smoke impurities, that one has enjoyed, without atmospheric obstructions, a place in the sun?

And here we touch one of the greatest deprivations caused by smoke pollution: the loss of sunlight. Dr. Wallace Wallin says that the smoke from factory and domestic fires, by filling the atmosphere with opaque clouds of smoke and by inducing mists and fogs, deprives city dwellers of "the luminous, vitalising, cheering, health-giving germicidal rays of the sun." How much, not only of positive disease, but of negative—depression, lowness of tone, irritability, and so forth—arises from this deprivation! It has been found by careful investigation that in an industrial centre no less than ten per cent. of the sunlight was cut off from passing through the last 100 feet of atmosphere. Ten per cent. of the most vivifying tonic lost to millions of our population through a preventable nuisance!

Of economic waste by smoke pollution much has been written. It is a great but not the greatest loss. It has been estimated as high as £4 per head per annum of an industrial population, and as low as £1. Obviously many millions per year are lost through this evil. Add the economic waste to the loss of health and efficiency and we have an appalling

**THE PUBLIC WELL-BEING**—(*continued*)

account against smoke pollution. How can it be remedied? Clearly there is work here for the State, the local authorities, and the individual. One thing the State could do immediately is to strengthen and apply more rigorously regulations against the unnecessary emission of smoke. A more distant reform would be to press on with the plan for central electrical generating schemes. Municipalities can aid inventions for household grates which minimise smoke production, and, in their own housing schemes, adopt those grates. They can also promote central hot-water systems wherever the conditions allow. By this latter method domestic smoke production in the warm months could be immensely reduced.

The part the individual may play in this work of reform depends upon the intensity of his interest; and, therefore, one advises him first, if so far he has been indifferent, to look into the remarkable facts which are sure to excite and stimulate an active interest.

